# Identification of Potential Biomarkers of Radiation Exposure in Blood Cells by Capillary Electrophoresis Time-of-Flight Mass Spectrometry

**DOI:** 10.3390/ijms21030812

**Published:** 2020-01-27

**Authors:** Lue Sun, Yohei Inaba, Norie Kanzaki, Mahesh Bekal, Koichi Chida, Takashi Moritake

**Affiliations:** 1 Health Research Institute, Department of Life Science and Biotechnology, National Institute of Advanced Industrial Science and Technology (AIST), Central 6, 1-1-1 Higashi, Tsukuba, Ibaraki 305-8566, Japan; 2 Course of Radiological Technology, Health Sciences, Tohoku University Graduate School of Medicine, 2-1 Seiryo, Aoba, Sendai, Miyagi 980-8575, Japan; 3 Department of Radiation Disaster Medicine, International Research Institute of Disaster Science, Tohoku University, Aramaki Aza-Aoba 468-1, Aoba-ku, Sendai 980-0845, Japan; 4 Ningyo-toge Environmental Engineering Center, Japan Atomic Energy Agency, 1550 Kamisaibara, Kagamino-cho, Tomata-gun, Okayama 708-0698, Japan; 5 Department of Radiological Health Science, Institute of Industrial Ecological Sciences, University of Occupational and Environmental Health, Japan, 1-1 Iseigaoka, Yahatanishi-ku, Kitakyushu, Fukuoka 807-8555, Japan

**Keywords:** radiation, metabolome, biodosimetry, blood cell, disaster medicine

## Abstract

Biodosimetry is a useful method for estimating personal exposure doses to ionizing radiation. Studies have identified metabolites in non-cellular biofluids that can be used as markers in biodosimetry. Levels of metabolites in blood cells may reflect health status or environmental stresses differentially. Here, we report changes in the levels of murine blood cell metabolites following exposure to X-rays in vivo. Levels of blood cell metabolites were measured by capillary electrophoresis time-of-flight mass spectrometry. The levels of 100 metabolites were altered substantially following exposure. We identified 2-aminobutyric acid, 2′-deoxycytidine, and choline as potentially useful markers of radiation exposure and established a potential prediction panel of the exposure dose using stepwise regression. Levels of blood cell metabolites may be useful biomarkers in estimating exposure doses during unexpected radiation incidents.

## 1. Introduction

Exposure to ionizing radiation during and following radiation and nuclear leaks and explosions may cause acute damage and increase the risk of developing chronic conditions such as cancer, cataracts, and dermatitis [1]. Biodosimetry is a useful tool for estimating personal exposure doses [2]. While the primary objective of biodosimetry is to guide medical intervention following exposure to radiation, the value of this technique extends to supporting epidemiological studies of long-term health risks [3]. Numerous biodosimetric markers have been reported, such as chromosomal aberrations [4], DNA damage [5], free radicals [6], and dysregulation of gene expression [7], antioxidant production [8], and metabolites [9].

Exposure to ionizing radiation affects metabolite concentrations. Abe et al. reported changes in urinary excretion of taurine in irradiated mice in 1968 [10]. More recently, metabolome analysis techniques have enabled the measurement of several dozen to several hundred metabolites simultaneously and are widely used in basic and clinical medical studies [11,12,13]. Numerous studies have measured metabolites following radiation exposure using metabolomic techniques [14], but these studies focused on non-cellular biofluids such as serum [15,16,17], plasma [18], urine [19,20], and saliva [21]. Only a few studies have investigated blood cells; although red blood cells lack nuclei and cellular organelles, they use glycolysis and the pentose phosphate pathway for ATP production and maintain redox homeostasis and osmoregulation [22]. Chaleckis et al. demonstrated that human age-related metabolites were enriched in blood cells [22], and the same group recently reported that the concentrations of a number of metabolites in plasma and blood cells change during fasting [23]. Thus, levels of metabolites in red blood cells may reflect health status or environmental stresses differentially than levels in plasma [22].

Previous studies have used nuclear magnetic resonance [24], gas chromatography–mass spectrometry (GC–MS) [25], or liquid chromatography–mass spectrometry (LC–MS) [26] to measure metabolite levels after irradiation, and only a few employed capillary electrophoresis–mass spectrometry (CE–MS). These techniques have varying sensitivities, specificities, and detection limits. Ramautar et al. analyzed human urine by CE–MS and LC–MS and found that CE–MS detected approximately 500 metabolites while LC–MS detected approximately 300 metabolites [27], and that the metabolite sets detected differed between techniques. The authors concluded that the CE–MS method was highly complementary to LC–MS by providing categorization by metabolite class.

In the present study, we used CE–MS to measure changes in the levels of blood cell metabolites in vivo following exposure to radiation. We applied total-body irradiation (1 or 3 Gy) to mice and collected blood cells 2 and 6 days after irradiation. A non-targeted metabolome analysis was performed using capillary electrophoresis time-of-flight mass spectrometry (CE–TOFMS). We were able to identify 100 metabolites whose levels were significantly altered following irradiation. We found that the levels of several metabolites involved in aspartic acid, urea, and creatinine metabolism and neurotransmitter-related metabolites decreased after exposure. We also found that 2-aminobutyric acid, 2′-deoxycytidine, and choline were potentially useful markers of exposure to ionizing radiation. To establish a prediction biomarker panel of the exposure dose, we performed stepwise regression and identified 10 metabolites for both measurement time points. These findings suggest that the levels of blood cell metabolites can be used as biodosimetric markers of exposure to radiation.

## 2. Results

### 2.1. Changes in the Levels of Blood Cell Metabolites Following Exposure to Ionizing Radiation

**Figure 1:**
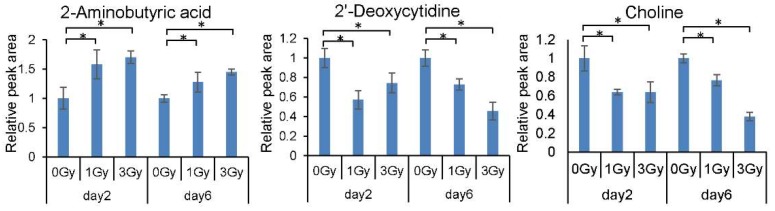
Dose and time response of levels of 2-aminobutyric acid, 2′-deoxycytidine, and choline in mouse blood cells. All quantitative data are means ± SD. * *p* < 0.05.

To identify blood cell radiation-responsive metabolites, we subjected C57BL/6J mice to irradiation (0, 1, or 3 Gy) and collected blood cells 2 and 6 days after exposure. Metabolome analysis was then performed by CE–TOF MS. We detected 306 annotated metabolites and 10 unknown peaks (Table S1). We found that the levels of 38 metabolites increased significantly after exposure, levels of 61 metabolites decreased, and the levels of one metabolite decreased and then increased (Table 1). Notably, the levels of 2-aminobutyric acid increased, and the levels of 2′-deoxycytidine and choline decreased in mice exposed to 1 and 3 Gy of ionizing radiation (Figure 1).

**Figure 2:**
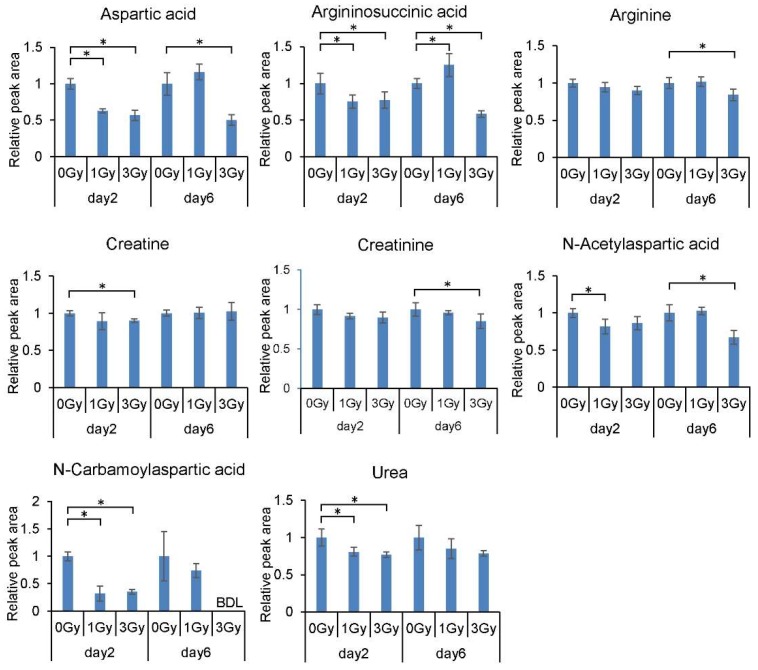
Dose and time response of levels of metabolites related to aspartic acid, urea, and creatinine metabolism in mouse blood cells. All quantitative data are means ± SD. * *p* < 0.05.

We found that the levels of several metabolites related to aspartic acid, urea, and creatinine metabolism decreased significantly following exposure to ionizing radiation (Figure 2).

**Figure 3:**
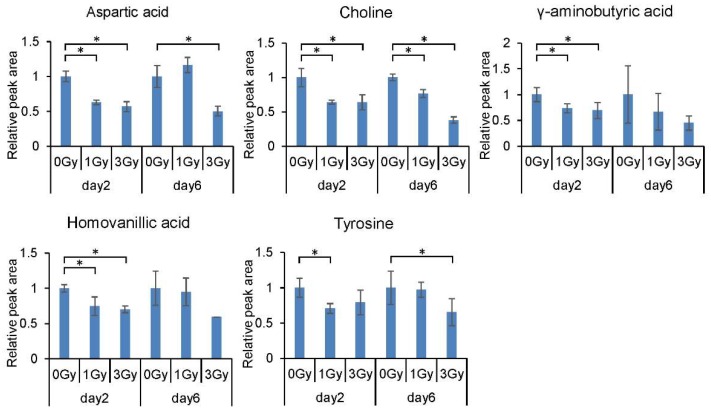
Dose and time response of levels of neurotransmitter-related metabolites in mouse blood cells. All quantitative data are means ± SD. * *p* < 0.05, Welch’s *t*-test.

We also found that the levels of several neurotransmitter-related metabolites decreased significantly following exposure to ionizing radiation, including those of aspartic acid, tyrosine, choline, homovanillic acid, and γ-aminobutyric acid (Figure 3).

### 2.2. Multivariate Analysis of Blood Cell Metabolites Following Exposure to Ionizing Radiation

**Figure 4:**
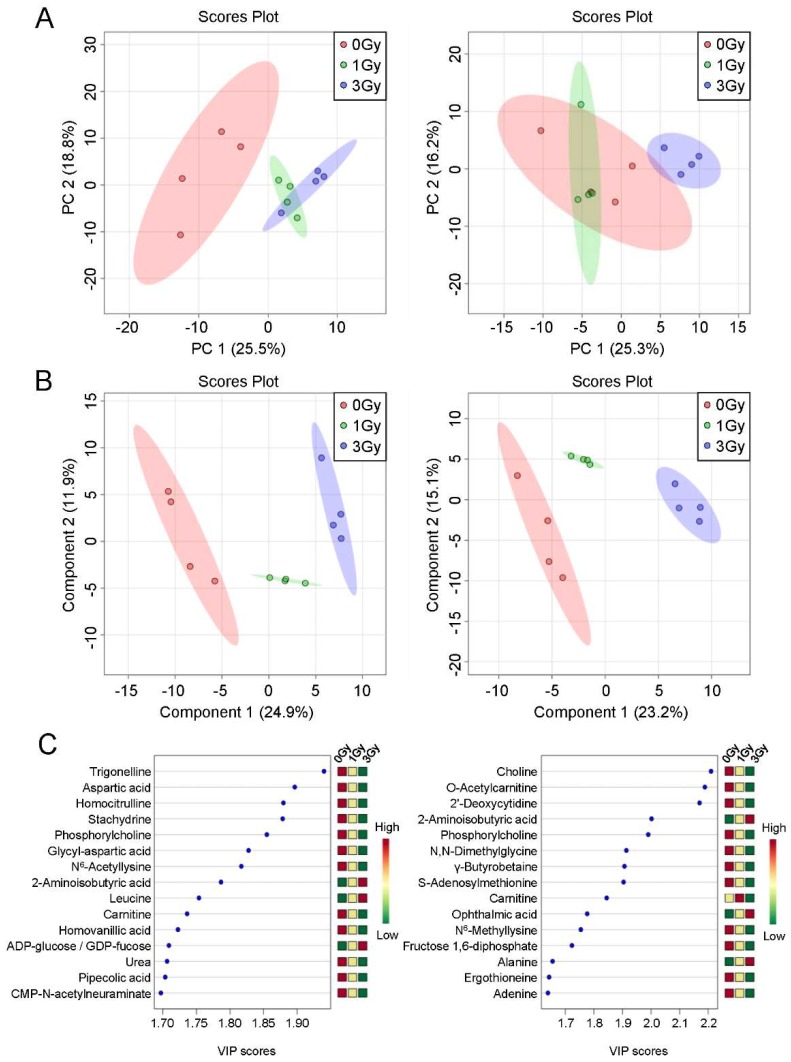
Multivariate data analysis of the blood cell metabolome from mice exposed to ionizing radiation. Principal component analysis (**A**) and partial least squares discriminant analysis (**B**) were performed. Circles indicate the 95% confidence interval. (**C**) Top 15 variable importance in projection (VIP) scores from partial least squares discriminant analysis of component 1. PC, principal component.

We performed principal component analysis (PCA) and partial least squares discriminant analysis (PLS–DA). PCA showed that the 95% confidence interval of the control group was separate from that of the mice exposed to 1 or 3 Gy of ionizing radiation, but the 95% confidence intervals of the two exposure groups overlapped on day 2 post-exposure. The 95% confidence intervals of all three groups overlapped on day 6 post-exposure (Figure 4A). PLS–DA clearly separated the 95% confidence intervals of the three groups on both day 2 and day 6 (Figure 4B). The top 15 PLS–DA variable importance in projection scores (Figure 4C) suggested that alterations in the levels of these metabolites were major contributors to class discrimination.

### 2.3. Establishment of a Potential Exposure Dose Prediction Panel

To establish a potential prediction panel of the exposure dose, we performed stepwise regression, which selected 10 metabolites from both measurement days whose levels differed significantly from levels in the controls and that had good prediction performance (Table 2).

## 3. Discussion

We previously reported that exposure to ionizing radiation decreases antioxidant capacity in whole blood but not in serum [8]. Thus, we hypothesized that the levels of blood cell metabolites would be altered by radiation to a greater extent than in serum or plasma. We irradiated mice with 1 and 3 Gy of X-rays and measured blood cell metabolites after 2 and 6 days. The threshold dose for mortality in human adults exposed acutely to ionizing radiation has been reported as 1 Gy [28], and exposure to 3 Gy has been shown to shorten lifespan in C57BL/6 mice [29]. The recommended time frame for performing biodosimetry assays and triage is within 2 days of exposure for individuals with physical injuries and within 6 days for 1,000,000 victims [30], guiding our choice of doses and measurement points.

We detected 306 metabolites and 10 unknown peaks and found that the levels of 100 of the metabolites increased or decreased significantly following exposure to ionizing radiation. Pannkuk et al. detected 112 compounds in serum from radiation–exposed rhesus monkeys analyzed by GC–MS [25], and most of those differed from the ones we found in the present study, with only 19 overlapping metabolites. The differences may be attributable to the analytical technique or the species studied. Both our study and a recent one in rhesus monkeys found a decrease in the levels of aspartic acid, glycine, and tyrosine [31], but while we observed an increase in the levels of valine and alanine and a decrease in the levels of carnitine, the other study reported opposing findings [31]. These differences may be attributable to blood cell uptake of amino acids to control metabolite levels [32] and suggest that co-analysis of metabolites in blood cells and in serum should increase the accuracy of biodosimetry.

We found an increase in the levels of 2-aminobutyric acid and decreases in the levels of 2′-deoxycytidine and choline following exposure to radiation, indicating that these metabolites are potentially useful markers of exposure to 1 Gy or more of ionizing radiation. To date, however, no studies have reported the use of theses metabolites in biodosimetry; 2′-deoxycytidine is a nucleoside component of DNA, consisting of cytosine and deoxyribose, and our findings suggest that radiation-induced DNA damage decreases its production. Choline deficiency induces oxidative stress [33], suggesting that radiation-induced decreases lead to the production of free radicals.

We found that the levels of several metabolites related to aspartic acid, urea, and creatinine metabolism decreased following exposure. The levels of aspartic acid decreased in serum following exposure to radiation, suggesting that aspartic acid may enter the citric acid cycle via catabolic pathways [34]. Interestingly, we found that the levels of several neurotransmitter-related metabolites (i.e., aspartic acid, tyrosine, choline, homovanillic acid, and γ-aminobutyric acid) decreased in the exposed groups. Levels of aspartic acid, tyrosine, and γ-aminobutyric acid have been reported to decrease in patients with depression [35,36], and ionizing radiation can induce behavioral aberrations and cognitive dysfunction in mice and humans receiving radiotherapy [37,38]. The threshold dose for cognitive defects is considered to be 1 Gy [28], and decreases in the levels of neurotransmission-related metabolites may be a mechanism of ionizing radiation-induced cognitive dysfunction.

We excluded metabolites whose levels were beneath the limits of detection and those that were not identified. Our PLS–DA analysis discriminated between exposure groups at the two measurement time points and suggested that the levels of blood cell metabolites are useful biomarkers of radiation exposure. However, the top 15 variable importance in projection scores differed between the two measurement time points, implying that the metabolic profile was altered substantially over time post-exposure. To assess the exposure dose, we performed stepwise regression, and our analysis suggested that the levels of blood cell metabolites can be useful in estimating exposure doses, but that prediction panels for use in biodosimetry should be time-dependent and vary by days from exposure.

Our study has three limitations. First, the sample number for each group (*n* = 4), dose (0, 1, and 3 Gy), and time points (two measurements) were small. Second, we collected blood at different times of day and thus were unable to compare day 2 and 6 measurements directly. To resolve these problems, absolute quantification is required. Third, we used mice, and do not know whether similar changes in metabolite levels occur in humans. Future studies should recruit patients who receive clinical radiotherapy, use absolute quantification, and both technical and intra- and inter-individual variations should be accounted for.

In summary, we identified three potential discriminating markers of exposure to ionizing radiation and established potential prediction panels of the exposure dose. Our findings may contribute to biodosimetry of radiation in humans and improve radiation disaster medicine.

## 4. Materials and Methods

### 4.1. Animals, Exposure, and Preparation of Blood Cells

Six-week-old male C57BL/6J mice were obtained from Japan SLC (Shizuoka, Japan). Their food and drinking water were sterilized by autoclaving. After at least 1 week of acclimation, mice were subjected to 0, 1, or 3 Gy of X-rays in total-body irradiation (150 kV, 20 mA, filter: 0.2 mm Cu and 0.5 mm Al, MBR-1520R-3, Hitachi Power Solutions, Ibaraki, Japan). The irradiation dose rate was 0.88 Gy/min. Four mice were used per group. Mouse whole blood was collected via cardiac puncture 2 or 6 days after exposure. Whole blood was centrifuged at 3000× *g* at 4 °C for 10 min to separate plasma from blood cells.

### 4.2. Metabolite Extraction

A blood cell suspension (200 µL) was collected by pipette and added to 1800 µL methanol containing internal standards (H3304-1002, Human Metabolome Technologies, Inc., Tsuruoka, Japan) at 0 °C to inactivate enzymes. The extract was thoroughly mixed with 2000 µL chloroform and 800 µL Milli-Q water (MilliporeSigma, Burlington, MA, USA) and centrifuged at 2300× *g* and 4 °C for 5 min. Next, 400 µL of the upper aqueous layer were centrifugally filtered through a Millipore 5-kDa cutoff filter to remove proteins. The filtrate was centrifugally concentrated and resuspended in 50 µL Milli-Q water for metabolome analysis.

### 4.3. Measurement of Blood Cell Metabolites by CE–TOFMS

Metabolome analysis was conducted using the Basic Scan package (Human Metabolome Technologies, Inc.) using CE–TOFMS and methods described previously [39,40]. Briefly, CE–TOFMS analysis was carried out using an Agilent CE capillary electrophoresis system equipped with an Agilent 6210 time-of-flight mass spectrometer, Agilent 1100 isocratic high-performance liquid chromatography pump, Agilent G1603A CE–MS adapter kit, and Agilent G1607A CE–ESI–MS sprayer kit (Agilent Technologies, Santa Clara, CA, USA). The systems were controlled by Agilent G2201AA ChemStation software version B.03.01 for CE (Agilent Technologies) and connected through a fused silica capillary (50-μm i.d. × 80-cm total length) with commercial electrophoresis buffer (H3301-1001, Human Metabolome Technologies, Inc.) as the electrolyte. The spectrometer was scanned from *m/z* 50 to 1000 [39]. Peaks were extracted using MasterHands automatic integration software (Keio University, Tsuruoka, Japan) to obtain peak information such as the mass-to-charge ratio, peak area, and migration time [41]. Signal peaks corresponding to isotopomers, adduct ions, and other product ions of known metabolites were excluded, and the remaining peaks were annotated according to the Human Metabolome Technologies metabolite database by their mass-to-charge ratio values and migration time. Areas of the annotated peaks were then normalized by internal standard levels and sample volumes to obtain the relative levels of each metabolite. Since the blood cell preparations were heterogeneous (white blood cells, red blood cells, and platelets), we did not normalize metabolite levels by blood counts.

### 4.4. Statistical Analysis

The mean and SD were calculated for each data point. One-way ANOVA and post-hoc Dunnett’s tests were used to analyze significant differences between groups using the R statistical package. A *p*-value < 0.05 was considered statistically significant. Metabolite pathway clusters and categories were derived from the Kyoto Encyclopedia of Genes and Genomes (KEGG) (https://www.genome.jp/kegg/) and the Human Metabolome Database (HMDB) (http://www.hmdb.ca/). PCA and PLS–DA were performed using MetaboAnalyst (https://www.metaboanalyst.ca/MetaboAnalyst/home.xhtml) [42]. Stepwise regression was performed using JMP software (SAS Institute, Cary, NC, USA). We excluded metabolites whose levels were below the detection limit and unidentified peaks. Data were normalized by mean centering and divided by the SD of each variable for PCA, PLS–DA, and stepwise regression.

### 4.5. Ethical Considerations

All animal experiments were performed in accordance with the Animal Care Guidelines of the University of Occupational and Environmental Health, Japan (UOEH.J.) All animal husbandry procedures and experiments were approved by the Animal Experiment Committee of UOEH.J. (permit number: AE15-009, approval date: 28/March/2017).

## Figures and Tables

**Table 1:** Changes in the levels of blood cell metabolites following exposure to ionizing radiation.

Metabolite	Category ^†^	Day 2		Day 6	
		Fold Change		Fold Change	
		1 Gy/0 Gy	3 Gy/0 Gy	1 Gy/0 Gy	3 Gy/0 Gy
Increased (38 metabolites)					
2-Aminobutyric acid		1.58 *	1.7 *	1.27 *	1.45 *
3-Phosphoglyceric acid	Sugar metabolism	1.33	1.71 *	1.05	0.74
5-Oxohexanoic acid		1.48 *	1.21	1.37 *	1.22
ADP-glucose	Nucleotide sugars, Nucleotide sugars, Vascular	1.22	1.42 *	1.08	1.07
GDP-fucose					
ADP-ribose		0.48	0.87	1.1	1.83 *
Alanine	Cytokine, Hormone, Hemocyte, Renal disease, Uremic toxin, Sugar metabolism	1.04	1.06	1.12	1.3 *
ATP	Purine bases, Sugar metabolism	1.22	1.46 *	1.09	0.81
Cumic acid		N.D.	N.D.	1.4 *	1.1
Decanoic acid	Lipid, Fatty Acid metabolism	1.25	1.01	1.38 *	1.06
Flavin mononucleotide		1.62	2.08 *	1.29 *	0.78
Glutamine	Hemocyte	0.99	1.15 *	1.05	1
Glutathione (GSH)	Anti-oxidant, Lipid Fatty Acid metabolism, Methylglyoxal	0.83	1.67 *	0.92	0.95
GTP	Purine bases	1.38	1.76 *	1.24	0.81
Heptanoic acid	Lipid, Fatty Acid metabolism	1.51	1	1.4 *	1.17
Hexanoic acid	Lipid, Fatty Acid metabolism	1.33	1.06	1.21 *	1.02
Histidine		1.06	1.18 *	1.01	1.02
Isoleucine	Essential amino acid, Lipid, Fatty Acid metabolism, Liver disease, Sugar metabolism	1.03	1.27 *	1.12	1.01
Isobutyric acid	Apoptosis, Carcinogenesis, Cell function, Lipid, Fatty Acid metabolism	1.67 *	1.11	1.08	1.12
Butyric acid					
Isovaleric acid	Lipid, Fatty Acid metabolism	1.57 *	1.16	1.19	1
Valeric acid					
Leucine	Essential amino acid, Insulin, Lipid, Fatty Acid metabolism, Liver disease, Protein metabolism	1.11	1.33 *	1.17	1.19 *
Malonylcarnitine		1.02	1.16	1.31 *	0.85
Octanoic acid	Lipid, Fatty Acid metabolism	1.02	0.94	1.15 *	0.98
Octanoylcarnitine		0.53	0.81	1.31 *	N.D.
Ophthalmic acid		1.11	1.16	1.15	1.34 *
p-Toluic acid		1.7 *	1.12	N.D.	N.D.
m-Toluic acid					
o-Toluic acid					
Pelargonic acid	Lipid, Fatty Acid metabolism	1.33	1.04	1.22 *	0.99
Phenylalanine	Catecholamines and Derivatives, Essential amino acid	1.04	1.17 *	1.06	0.92
Phosphoenolpyruvic acid	Sugar metabolism	1.45	1.9 *	1.13	0.75
*S*-Methylcysteine	Anti-oxidant, Sugar metabolism	0.97	1.17 *	N.D.	N.D.
Sedoheptulose 7-phosphate		1.44	1.24	1.19	1.63*
Thiaproline		1.13	1.52 *	1.15	1.09
Tyramine	Catecholamines and Derivatives, Nervous system	N.D.	N.D.	1.47 *	1.17
UDP-*N*-acetylgalactosamine-1		1.22 *	1.27 *	1.09	0.93
UDP-*N*-acetylglucosamine-1					
Undecanoic acid	Lipid, Fatty Acid metabolism	N.D.	N.D.	1.56 *	1.09
Valine	Essential amino acid, Liver disease, Nervous system, Sugar metabolism	1.06	1.25 *	1.06	1.02
XA0002 (unknown peak)		0.93	0.97	0.92	1.16 *
XC0132 (unknown peak)		1.13	1.26 *	N.D.	N.D.
γ-Glutamyl-cysteine		1.03	2.06 *	0.83	0.87
Decreased (61 metabolites)					
2-Hydroxy-4-methylvaleric acid	Maple syrup urine disease	0.63 *	0.6 *	0.78	0.8
2’-Deoxycytidine	Pyrimidine bases	0.57 *	0.74 *	0.73 *	0.46 *
3-Indoxylsulfuric acid	Renal disease, Uremic toxin, Vascular	0.76	0.51 *	0.61	0.78
5’-Deoxy-5’-methylthioadenosine	Purine bases	0.84	0.55 *	0.9	0.89
7,8-Dihydrobiopterin	Vascular	0.41 *	0.39 *	0.99	0.3 *
Acetoacetamide		0.8	0.65 *	N.D.	N.D.
Adenine	Purine bases, Salvage pathway, Purine bases, Salvage pathway	1.32	1.09	0.91	0.71 *
Arginine	Guanidino compounds	0.94	0.9	1.02	0.84 *
Aspartic acid	Nervous system, Neuropsychiatric disorder, Sugar metabolism	0.63 *	0.57 *	1.16	0.5 *
Betaine	Osmolytes, Renal disease, Uremic toxin, Transmethyration	0.79 *	0.76 *	1.11	0.83
Betonicine		0.71 *	0.69 *	0.86	N.D.
Butyrylcarnitine		0.73	0.63 *	0.75	0.93
Carnitine	Lipid, Fatty Acid metabolism, Liver disease, Cardiac disease	0.74 *	0.72 *	1.02	0.55 *
Choline	Lipid, Fatty Acid metabolism, Transmethyration	0.64 *	0.64 *	0.77 *	0.38 *
Citrulline		0.87	0.8 *	1.04	0.92 *
CMP-*N-*acetylneuraminate	Nucleotide sugars	0.45 *	0.44 *	1.01	0.95
Creatine	Cell function	0.89	0.9 *	1.01	1.03
Creatinine	Protein metabolism, Renal disease, Uremic toxin,	0.92	0.9	0.95	0.85 *
Ectoine		0.98	0.61 *	0.84	0.67
Ergothioneine	Anti-oxidant, Oxidative stress	0.98	1.03	0.95	0.69 *
Ethanolamine		0.79 *	0.89	1.08	0.59
Fructose 1,6-diphosphate	Sugar metabolism	0.75	0.92	0.87	0.4 *
γ-Aminobutyric acid	Nervous system, Sugar metabolism	0.73 *	0.69 *	0.67	0.45
Glycine		0.84 *	0.92	1.13	0.85
Glycyl-aspartic acid		0.46 *	0.41 *	1.3	0.36 *
Glycerol 3-phosphate	Sugar metabolism	0.62 *	0.69 *	1.05	0.91
Hippuric acid		0.98	0.57 *	0.63	1.2
Homocitrulline		0.84	0.65 *	0.76 *	0.79
Homovanillic acid	Catecholamines and Derivatives, Dopamine related substances, Nervous system	0.74 *	0.7 *	0.95	0.6
Imidazolelactic acid		0.64 *	0.85	0.85	0.71
Isethionic acid		0.63 *	0.63 *	0.87	0.87
Methionine sulfoxide	Oxidative stress	0.9	0.67 *	0.95	1.13
*N*-Acetylaspartic acid	*N-*Acetylated compounds, Nervous system	0.82 *	0.86	1.03	0.67 *
*N-*Acetylgalactosamine	Sugar metabolism	0.91	0.99	1.08	0.69 *
*N-*Acetylmannosamine					
*N-*Acetylglucosamine					
*N-*Carbamoylaspartic acid		0.32 *	0.35 *	0.74	N.D.
*N-*Methylproline		0.94	0.77 *	0.73	0.53
*N*,*N-*Dimethylglycine	Methylated compounds, Oxidative stress, Transmethyration	0.96	0.87	0.9	0.8 *
N6-Acetyllysine	Apoptosis, Cell function, DNA damage, *N-*Acetylated compounds	0.74 *	0.55 *	0.78	0.75 *
N6-Methyllysine	Methylated compounds, Transmethyration	0.97	1.02	0.95	0.78 *
O-Acetylcarnitine	Lipid, Fatty Acid metabolism, Nervous system	0.89	0.94	0.85 *	0.61 *
Phosphorylcholine	Liver disease	0.4 *	0.32 *	0.82	0.34 *
Pipecolic acid	Liver disease	0.81 *	0.77 *	0.86	0.75
Pyruvic acid	Ketosis, Lipid, Fatty Acid metabolism, Protein metabolism, Sugar metabolism	0.72 *	0.91	1.19	0.96
Ribulose 5-phosphate		0.58 *	0.69	0.89	0.75
*S*-Adenosylmethionine	Liver disease, Neuropsychiatric disorder, Oxidative stress, Transmethyration	0.94	1	0.94	0.87 *
*S*-Lactoylglutathione	Methylglyoxal	0.58 *	0.76	0.68	0.57
Symmetric dimethylarginine	Methylated compounds, Protein metabolism, Renal disease, Uremic toxin, Transmethyration, Vascular	0.91	1	1.05	0.6 *
Spermidine	Polyamines	0.3 *	0.41 *	1.18	0.11 *
Stachydrine	Osmolytes, Renal disease, Uremic toxin, Transmethyration	0.73 *	0.61 *	0.98	0.83
Thymidine	Pyrimidine bases	0.56 *	0.77 *	0.88	0.43 *
Trigonelline		0.83 *	0.62 *	0.88	0.96
Trimethylamine *N-*oxide	Osmolytes, Renal disease, Uremic toxin	0.69	0.55 *	1.52	1.36
Tyrosine	Catecholamines and Derivatives	0.71 *	0.79	0.97	0.65 *
Urea	Protein metabolism	0.81 *	0.77 *	0.85	0.78
XA0013 (unknown peak)		0.65	0.42 *	N.D.	N.D.
XA0033 (unknown peak)		0.45 *	0.39 *	N.D.	N.D.
XA0055 (unknown peak)		0.73	0.91	0.79	0.43 *
XC0040 (unknown peak)		0.77 *	0.78 *	0.83	0.68 *
XC0061 (unknown peak)		0.59 *	0.57 *	1.16	0.41 *
β-Alanine		0.93	0.79	1.05	0.57 *
γ-Butyrobetaine		0.76 *	0.78 *	0.78	0.36*
Increased and decreased (one metabolite)					
					
Argininosuccinic acid	Renal disease, Uremic toxin	0.75 *	0.77 *	1.25 *	0.58 *

* Comparative analysis was performed by one-way ANOVA and post hoc Dunnett’s test. A *p*-value of less than 0.05 was considered statistically significant. ^†^ Metabolite categories were derived from the Kyoto Encyclopedia of Genes and Genomes (KEGG) and the Human Metabolome Database (HMDB).

**Table 2:** Results of stepwise regression analysis.

Metabolite	Coefficient	Standard Error	*T*-Value	*p*-Value
Two days after irradiation (*R*^2^ = 1; *p* < 0.001; *F*-value = 4.76 × 10^10^)				
Constant	1.333	0.000000065	20,573,133	<0.001
Trigonelline	−0.731	0.000000704	−1,037,888	<0.001
γ-Glutamyl-cysteine	0.842	0.000000709	1,186,369	<0.001
Kynurenine	−0.382	0.000000265	−1,441,436	<0.001
Isethionic acid	−0.286	0.000000521	−548,634	<0.001
UDP-glucuronic acid	−0.181	0.000000773	−234,621	<0.001
Hypotaurine	−0.042	0.000000209	−202,910	<0.001
*N*^6^-Acetyllysine	0.029	0.000001165	24,655	<0.001
NADPH_divalent	0.005	0.000000767	6794	<0.001
*S*-Methylcysteine	−0.0002	0.000000627	−333	0.0019
Adenine	−0.00005	0.000000725	−72	0.0089
Six days after irradiation (*R*^2^ = 1; *p* < 0.001; *F*-value = 1.1 × 10^11^)				
Constant	1.333	0.00000120	1,109,219	<0.001
Choline	−1.180	0.00000631	−186,890	<0.001
Dihydroxyacetone phosphate	−0.115	0.00000407	−28,344	<0.001
Histamine	0.095	0.00000211	45,188	<0.001
Glycerophosphocholine	−0.129	0.00000559	−23,197	<0.001
Ornithine	0.092	0.00000387	23,692	<0.001
Fructose 1,6-diphosphate	0.063	0.00000397	15,820	<0.001
Ethanolamine	−0.017	0.00000413	−4103	<0.001
Methionine sulfoxide	0.004	0.00000381	1075	<0.001
Threonic acid	0.002	0.00000447	346	0.0018
Spermidine	−0.0004	0.00000684	−67	0.0094

## Supplementary Materials

Supplementary materials can be found at https://www.mdpi.com/1422-0067/21/3/812/s1.
